# Hunger among Inuit children in Canada

**DOI:** 10.3402/ijch.v72i0.20324

**Published:** 2013-04-23

**Authors:** Leanne C. Findlay, Kellie A. Langlois, Dafna E. Kohen

**Affiliations:** Health Analysis Division, Statistics Canada, Ottawa, Canada

**Keywords:** Inuit, hunger, children, social determinants, Aboriginal Children's Survey

## Abstract

**Background and objectives:**

Inuit populations may be at increased risk for experiencing poor nutrition or hunger due to limited access and availability to food. The prevalence and correlates of parental perceptions of hunger among a nationally representative sample of Inuit children in Canada have not yet been reported.

**Design:**

Data are from the 2006 Aboriginal Children's Survey (ACS). Sociodemographic information, dietary behaviours and hunger status were parent-reported via a household interview for Inuit children aged 2–5 years (n=1,234). Prevalence of hunger was calculated among Inuit children by sociodemographic factors and by dietary behaviours. In addition, a multivariate logistic regression model was conducted to determine factors associated with parental perception of ever experiencing hunger.

**Results:**

The prevalence of Inuit children in Canada aged 2–5 years ever experiencing hunger was 24.4%. Children who were reported to have experienced hunger consumed milk and milk products (p<0.001); fish, eggs and meat (p<0.05); fruits (p<0.001); and vegetables (p<0.001) significantly less often than never-hungry children. Fast food and processed foods, soft drinks and juice, and salty snacks, sweets and desserts were consumed as often as never-hungry children (all p>0.05). The majority (81%) of Inuit parents/guardians of ever-hungry children sought help from family or friends. Factors associated with an increased likelihood of experiencing hunger include sociodemographic characteristics (such as income and household size), living in an Inuit region and living in a community with cultural activities.

**Conclusion:**

About 1 in 4 Inuit children were reported by their parents to have experienced hunger, and hunger was associated with region, sociodemographic and community factors. Future research could further examine the impact of ever experiencing hunger on the health status of Inuit children and their families in Canada.

Food is a basic necessity of life; without it, one cannot be healthy. Among children in particular, healthy food choices and dietary behaviours are essential for proper growth and development. It is also possible that hunger (which is often labelled as severe food insecurity) (1) is associated with a decreased likelihood of meeting nutritional requirements. Hunger among children has been shown to be associated with poor health status (2), an increased number of chronic health conditions, anxiety and depression (3), increased developmental risk in the preschool period (4) and reduced academic performance and increased psychosocial difficulties (5, 6).

Constrained access, availability and quality of food are important issues for Inuit people, especially in remote regions (7). Fruits and vegetables in particular are not widely available in northern communities (8) and can be expired or of suboptimal quality due to transportation logistics (7). Additionally, the cost of ‘market’ foods (i.e. those purchased in a grocery store) in northern communities is substantially higher than those in the south (9, 10) as the cost of transportation increases the price of food (11). Traditional food consumption, such as game animals, may satisfy some dietary requirements although there is some evidence to suggest that traditional food consumption may be declining due to shifts in hunting (11, 12) and climate change (13). However, traditional food consumption represents a relatively small portion of Inuit children's total caloric intake (e.g. less than 3% of total daily calories among Inuit children in Nunavik) (14).

In addition to the issues of accessibility and availability, the Inuit population may be at higher risk for experiencing food insecurity and hunger. Hunger is often considered an extreme manifestation of food insecurity (1, 2). Approximately 3% of Canadian children in general have experienced hunger (2). Among Inuit pre-schoolers living in Nunavut, Egeland et al. (15) found that almost 70% lived in food insecure households. Those who lived in a food insecure household were also more likely to have consumed traditional foods (16). Children in Nunavut have been shown to be more at risk of food insecurity than those living in the Inuvialuit or Nunatsiavut regions (17). However, to our knowledge no study has shown the prevalence of hunger among Inuit children for each Inuit region in Canada. In addition, little research has examined the diets of Inuit children who experience hunger, including traditional food consumption.

There are a limited number of studies that examine the correlates of hunger and/or food insecurity, particularly among children. In a longitudinal study of Canadian children in general (i.e. not Aboriginal specific), the odds of experiencing hunger were higher with increased child age, greater number of children in the home and ever having lived in a rented dwelling (as opposed to owning); odds were lower with increasing household income (2). Furthermore, it has been shown that families who experience multiple moves (2 or more times in the previous year) are at increased risk for food insecurity (18). Specific to the Inuit population, the Nunavut Inuit Health Survey found that children in food-insecure homes had a greater prevalence of household crowding and reporting of income support. However, the number of adults in the home was associated with a decreased risk for food insecurity (16). In general, it has been suggested that Inuit who are most vulnerable to food insecurity are those who are the most socially and economically disadvantaged, including single mothers, those with low levels of education and individuals with substance abuse issues (e.g. smoking or drugs) (12, 19). Those who are engaged and participate in the community, perhaps through food sharing traditional foods, may be less at risk for hunger (17), suggesting that community or cultural involvement may be an important factor in understanding the dynamics of hunger for Inuit children.

The purposes of the current study are threefold. First, this study aims to report the prevalence of experiencing hunger among a nationally representative sample of 2–5-year-old Inuit children in Canada. The tool used in this study reflected a parent-reported measure of hunger status only (not food insecurity). Second, dietary behaviours, including the consumption of foods included within the Canada Food Guide (20) as well as other commonly consumed foods and traditional or country foods, were described in terms of child hunger status. Finally, correlates of hunger for Inuit children were explored in a multivariate logistic regression model.

## Methods

### Data source

The data were from the 2006 Aboriginal Children's Survey (ACS) which was jointly conducted by Statistics Canada and Human Resources and Skills Development Canada. Designed to provide data about children's early development and their social and living conditions, the ACS was developed by Statistics Canada and Aboriginal advisors from across the country. The target population consisted of First Nations children not living on reserve, Métis children and Inuit children in the 10 provinces, as well as all children living in the 3 territories.

For the survey, a sample of children younger than age 6 was selected from households with children identified by the 2006 Census as having Aboriginal ancestors; and/or identified as North American Indian and/or Métis and/or Inuit; and/or had treaty or registered Indian status; and/or had Indian Band membership. More information on the ACS is available elsewhere (21). For the current study, only those children who were identified as Inuit (single or multiple identity) were considered. In addition, only those who were aged 2–5 years were included (n=1,234) as the outcome measure was asked only of this age group.

**Fig. 1:**
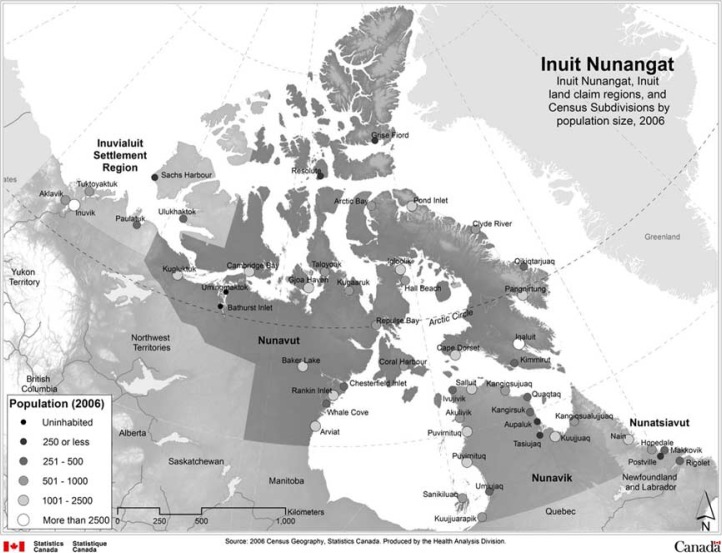
Map of Inuit Nunangat, 2006. Source: Peters PA. 2012. Shifting transitions: health inequalities in Inuit Nunangat in perspective. J Rural Community Dev. 7:36–58. With permission.

The parent/guardian provided information about the child's sociodemographic characteristics, including: child age, gender and Aboriginal identity, parental education (no parent who had graduated from high school, 1 parent with high school graduation or more, 2 parents with high school graduation or more), number of people raising the child (1, 2, or 3 or more), household size, household income, number of times the child had moved, and home ownership (vs. rent). Inuit children were identified as living in Inuit Nunangat (which includes the 4 northern land regions of Nunatsiavut, Nunavik, Nunavut, Inuvialuit) or outside Inuit Nunangat (see Fig. 1 for a map of the Inuit regions). The parent indicated whether or not there was a smoker living in the household and whether or not the respondent parent was looking for work. Parents were also asked about their satisfaction with their support network (very satisfied/satisfied vs. dissatisfied/very dissatisfied), and how they felt about the community as, (a) a place with actively involved members, and (b) a place with cultural activities (excellent/very good/good versus fair/poor).

To identify hunger status, respondent parents were asked if the child had “ever experienced being hungry because the family has run out of food or money to buy food”. Other studies have used this single-item indicator (2, 22). Further, Egeland et al. (23) found in the Inuit Child Health Survey that 90% of parents in severely food-insecure homes (in Nunavut) responded affirmatively that their children were ever hungry but they could not afford more food. In the ACS, of those who reported yes to the hunger question, questions were asked about the frequency of experiencing hunger (“occasionally”, “every few months”, “regularly at the end of each month”, or “more often than at the end of each month”) and coping strategies used to feed the child. Coping strategies included seeking help from family or friends, skipping meals or cutting back on the variety of foods consumed, and seeking help from institutions such as emergency food programs.

Children's dietary behaviours were reported by asking respondent parents how often the child usually ate the following foods and beverages: milk and milk products (such as yogurt, cheese, soy milk, or formula); fish, eggs and meat, such as beef, pork, or poultry; fast food and processed foods; fruit (excluding juice); vegetables; soft drinks and juice; and salty snacks, sweets and desserts. Respondents were also asked if the child ate any traditional or country foods such as bannock,^1^ large game, small game, sea-based mammals, or fish.

### Analysis

Frequencies of experiencing hunger as well as coping with hunger were calculated. Statistical comparisons were performed using chi-square tests and t-tests to determine differences in children's dietary consumption based on ever-hungry and never-hungry status (dichotomous outcome to be consistent with other research) (2, 22). Finally, a multivariate logistic regression model was conducted to determine factors independently associated with hunger (i.e. over and above other sociodemographic factors). Survey sampling weights were applied to account for the complex survey design and to render the analyses representative of the Inuit child population in Canada. Finally, a bootstrapping technique was applied when calculating estimates of variance (23).

## Results

The prevalence of Inuit children in Canada ever experiencing hunger was 24.4%. More specifically, 8% experienced hunger “occasionally”, 4% experienced hunger “every few months”, 7% experienced hunger “regularly at the end of each month” and 5% experienced hunger “more often than at the end of each month”. Of those Inuit children who had experienced hunger, 81% of parents/guardians reported that they had sought help from family or friends. Almost 1 in 4 (23%) ever-hungry Inuit children had parents/guardians who skipped meals or had their child(ren) skip meals or cut back on the variety of foods consumed, and 13% of ever-hungry Inuit children had parents/guardians who sought help from institutions such as emergency food programs.

In terms of regional findings, outside the 4 Inuit regions, almost 97% of Inuit children had never experienced hunger. However, within the 4 Inuit Nunangat regions, 32% had experienced hunger. More specifically, 33% of children living in Nunavik and in Nunavut, 22% of those living in Nunatsiavut and 11% of Inuit children living in the Inuvialuit region were reported to have ever been hungry.

Descriptive characteristics of the sample by hunger status are shown in Table I. Ever-hungry Inuit children were slightly older, had parents with lower levels of education, had experienced a greater number of moves, had a greater household size and lived in households with lower mean income than never-hungry children. Ever-hungry children were also more likely to live in a home that was rented (vs. owned), were more likely to live in a household with a smoker, and, although the majority were satisfied with their support network, on average ever-hungry children were less likely to have parents who were satisfied with their support network than never-hungry children.

**Table I:** Descriptive characteristics of Inuit children aged 2–5 by hunger status (never- versus ever-hungry), 2006

	Never-hungry (*n*=855)				Ever-hungry (*n*=310)			
			
Characteristics	%	95% CI	Mean	SE	%	95% CI	Mean	SE	χ^2^/t	p
Sex										
Female	48.3	(44.7–51.9)			50.6	(45.5–55.6)			0.52	>0.05
Male	51.7	(48.1–55.3)			49.4	(44.4–54.5)				
Age (years)			3.6	0.0			3.8	0.1	−4.59	<0.001
Parent education										
No parent with at least a high school education	41.4	(38.0–44.9)			75.1	(70.3–79.3)			55.62	<0.001
One parent with at least a high school education	34.0	(30.8–37.5)			19.0	(15.3–23.4)				
Two parents with at least a high school education	24.6	(21.2–28.3)			5.9^E^	(4.0–8.7)				
Number of people raising the child										
One	7.5	(5.8–9.6)			10.3	(7.7–13.7)			1.44	>0.05
Two	26.8	(23.4–30.5)			24.1	(19.8–29.0)				
Three or more	65.7	(61.9–69.3)			65.5	(60.4–70.3)				
Number of moves (per year of age)			1.7	0.0			2.2	0.1	−5.16	<0.001
Parent/guardian currently looking for work										
No	96.4	(94.8–97.5)			93.9	(91.1–95.8)			3.36	>0.05
Yes	3.6^E^	(2.5–5.2)			6.1^E^	(4.2–8.9)				
Household size			4.3	0.0			5.1	0.1	−8.34	<0.001
Household income (divided by $10,000)			5.5	0.1			4.0	0.1	10.44	<0.001
Home ownership										
No	69.9	(66.1–73.4)			89.9	(85.2–93.2)			51.53	<0.001
Yes	30.1	(26.6–33.9)			10.1^E^	(6.8–14.8)				
Smoker in the household										
No	83.4	(80.7–85.8)			70.7	(65.5–75.4)			19.55	<0.001
Yes	16.6	(14.2–19.3)			29.3	(24.6–34.5)				
Support network, support from family, friends, others										
Dissatisfied/very dissatisfied	7.1	(5.2–9.5)			12.5	(9.1–16.8)			5.52	<0.05
Very satisfied/satisfied	92.9	(90.5–94.8)			87.5	(83.2–90.9)				
Actively involved members of the community										
Fair/poor	25.0	(21.9–28.4)			28.6	(24.1–33.6)			1.52	>0.05
Excellent/very good/good	75.0	(71.6–78.1)			71.4	(66.4–75.9)				
Community as a place with First Nations, Métis and Inuit cultural activities										
Fair/poor	38.9	(35.2–42.7)			36.6	(31.3–42.2)			0.48	>0.05
Excellent/very good/good	61.1	(57.3–64.8)			63.4	(57.8–68.7)				

Source: Aboriginal Children's Survey 2006, Statistics Canada.

E Use with caution (co-efficient of variation 16.6% to 33.3%).

Table II shows the dietary behaviours of Inuit children by hunger status. Ever-hungry children were less likely to consume milk and milk products; fish, eggs and meat; fruit; and vegetables than never-hungry children. There were no differences by hunger status in the consumption of fast food or processed foods; soft drinks or juices; or salty snacks, sweets or desserts.

**Table II:** Dietary habits of Inuit children aged 2–5 by hunger status, 2006

		Never-hungry (*n*=855)		Ever-hungry (*n*=310)			
	
Dietary product	Frequency	%	95% CI	%	95% CI	χ^2^	p
Milk and milk products	Less than once per day	11.4	(9.5–13.6)	20.7	(16.8–25.2)	7.58	<0.001
	Once per day	16.3	(13.9–19.0)	16.2	(12.5–20.7)		
	Twice or more per day	72.3	(69.2–75.2)	63.1	(57.7–68.2)		
Fish, eggs and meat	Less than once per day	31.8	(28.6–35.2)	38.9	(33.9–44.1)	5.25	<0.05
	Once per day or more	68.2	(64.8–71.4)	61.1	(55.9–66.1)		
Fruit (excluding juice)	Less than once per day	27.6	(24.6–30.8)	43.5	(38.3–48.9)	12.91	<0.001
	Once per day	50.3	(46.5–54.2)	41.8	(36.5–47.3)		
	Twice or more per day	22.1	(18.7–25.8)	14.7	(11.5–18.6)		
Vegetables	Less than once per day	43.0	(39.4–46.6)	61.5	(56.1–66.7)	15.63	<0.001
	Once per day	47.5	(43.9–51.1)	32.6	(27.6–38.1)		
	Twice or more per day	9.5	(7.4–12.2)	5.9	(3.8–8.9)		
Fast food and processed food	Less than once per week	30.8	(27.4–34.4)	27.3	(22.9–32.2)	1.32	>0.05
	At least once per week	69.2	(65.6–72.6)	72.6	(67.8–77.1)		
Bread and pasta	Less than once per day	23.6	(20.9–26.5)	33.2	(28.6–38.2)	6.16	<0.01
	Once per day	36.1	(32.7–39.7)	28.5	(23.8–33.8)		
	Twice or more per day	40.3	(36.6–44.2)	38.2	(33.3–43.4)		
Soft drinks and juice	Less than once per day	21.4	(18.3–24.8)	21.0	(16.9–25.8)	1.15	>0.05
	Once per day	20.3	(17.5–23.5)	16.5	(12.7–21.3)		
	Twice or more per day	58.3	(54.5–62.0)	62.4	(56.9–67.6)		
Salty snacks, sweets and desserts	Less than once per week	9.1	(7.1–11.6)	8.8^E^	(6.3–12.4)	0.01	>0.05
	Once or more per week	35.6	(31.9–39.4)	35.5	(30.8–40.7)		
	Once per day or more	55.3	(51.4–59.2)	55.5	(50.3–60.7)		

Source: Aboriginal Children's Survey 2006, Statistics Canada.

E Use with caution (co-efficient of variation 16.6% to 33.3%).

A higher proportion of ever-hungry children consumed traditional foods as opposed to never-hungry Inuit children (Table III). Twice as many ever-hungry children consumed bannock at least once per day compared to their never-hungry counterparts. Significantly more ever-hungry children consumed large game, sea-based mammals, and salt and fresh water fish. There were no significant differences in the consumption of small game animals or game birds by hunger status.

**Table III:** Traditional foods consumed by Inuit children aged 2–5, by hunger status, 2006

		Never-hungry (*n*=855)		Ever-hungry (*n*=310)			
	
Dietary product	Frequency	%	95% CI	%	95% CI	χ^2^	p
Traditional foods	No	12.5	(9.5–16.2)	F	F	24.55	<0.001
	Yes	87.5	(83.8–90.5)	97.5	(94.5–98.9)		
Bannock or fry bread	Does not eat bannock	23.2	(19.5–27.4)	7.8^E^	(5.2–11.4)	25.00	<0.001
	Less than once per week	23.9	(20.8–27.3)	14.3	(10.8–18.7)		
	At least once per week	33.7	(30.4–37.1)	37.2	(32.2–42.4)		
	At least once per day	19.2	(16.9–21.8)	40.7	(35.5–46.1)		
Large game animals such as deer, moose or caribou	Never or less than once per month	27.9	(24.2–32.0)	11.1	(8.0–15.2)	36.37	<0.001
	Once per month or more	72.1	(68.0–75.8)	88.9	(84.8–92.0)		
Small game animals such as rabbit or muskrat	Never or less than once per month	94.0	(92.3–95.3)	94.2	(90.6–96.4)	0.01	>0.05
	Once per month or more	6.0	(4.7–7.7)	5.8^E^	(3.6–9.4)		
Game birds such as goose, duck, partridge or ptarmigan	Never or less than once per month	68.1	(64.9–71.1)	64.8	(59.7–69.6)	1.11	>0.05
	Once per month or more	31.9	(28.9–35.1)	35.2	(30.4–40.3)		
Sea-based mammals such as whale, seal or walrus	Does not eat	51.9	(48.2–55.6)	29.1	(24.6–34.0)	45.32	<0.001
	Less than once per month or more	48.1	(44.4–51.8)	70.9	(66.0–75.4)		
Salt and fresh water fish	Never or less than once per month	34.3	(30.5–38.3)	27.1	(22.4–32.2)	5.16	<0.05
	Once per month or more	65.7	(61.7–69.5)	72.9	(67.8–77.6)		

Source: Aboriginal Children's Survey 2006, Statistics Canada.

E Use with caution (co-efficient of variation 16.6% to 33.3%).

F Estimate not provided because of extreme sampling variability or small sample size.

Table IV shows the results of a multivariate logistic regression examining the odds of ever experiencing hunger. Factors independently associated with increased odds of experiencing hunger include having 1 parent who had a high school education or more (compared to 2 parents with a high school education or more), and greater household size. The odds of experiencing hunger were lower with increasing household income, home ownership, and if the parent felt that the community was a place with cultural activities. Traditional food consumption was not associated with ever being hungry once the other factors were considered. However, living in one of the Inuit regions was associated with hunger: Inuit children who lived in Nunatsiavut, Nunavik, Nunavut, or the Inuvialuit region had increased odds of experiencing hunger compared to those living outside Inuit Nunangat.

**Table IV:** Factors predicting parent-reported ever being hungry, Inuit children aged 2–5

Predictor variable	OR	95% CI
Sex		
Male	0.84	0.61–1.14
Female	1.00	
Child age	0.96	0.84–1.10
Parent education		
No parent with high school education	0.99	0.54–1.83
One parent with high school education	1.96*	1.09–3.53
Two parents with high school education	1.00	
Number of people raising the child		
Raised by 1 person	0.96	0.53–1.75
Raised by 2 people	1.00	
Raised by 3+ people	0.84	0.57–1.25
Household size	1.22*	1.12–1.33
Household income	0.84*	0.80–0.89
Number of moves	1.05	0.94–1.17
Home ownership		
Yes	0.53*	0.33–0.86
No	1.00	
Smoker in the household		
Yes	1.19	0.80–1.78
No	1.00	
Support network		
Very satisfied or satisfied	0.68	0.39–1.21
Dissatisfied or very dissatisfied	1.00	
Community with actively involved members		
Excellent/very good/good	0.93	0.63–1.38
Fair/poor	1.00	
Community with cultural activities		
Excellent/very good/good	0.69*	0.49–0.99
Fair/poor	1.00	
Eats traditional foods		
Child eats traditional foods	0.44	0.16–1.25
Does not eat traditional foods	1.00	
Parent/guardian looking for work		
Yes	1.41	0.68–2.92
No	1.00	
Inuit region		
Nunatsiavut	18.48*	7.81–43.72
Nunavik	29.80*	13.11–67.74
Nunavut	23.14*	10.37–51.68
Inuvialuit	4.61*	1.66–12.80
Outside Inuit Nunangat	1.00	

Source: Aboriginal Children's Survey 2006, Statistics Canada.

* Significant at p≤0.05.

## Discussion

Statistics Canada's Aboriginal Children's Survey provides a first look at the national prevalence of hunger among Inuit children in Canada. Our findings indicate that parents of one-quarter of Inuit children in Canada reported that their child had ever experienced hunger, which is higher than the reported prevalence of hunger among Canadian children overall (at 3.3%) (2). The proportion of Inuit children who had ever experienced hunger, by region, was: 33% in Nunavut and in Nunavik, 22% in Nunatsiavut, 11% in Inuvialuit and 3% outside Inuit Nunangat. Another study on Inuit pre-schoolers (aged 3–5 years, living in Nunavut) found the prevalence of severe food insecurity, often equated with hunger, to be 34% (24). Results from the International Polar Year Inuit Health Survey (2007–2008) also found that 26% of children in Nunavut, 11% of those in Nunatsiavut and 6% of those in Inuvialuit were severely food insecure (17). Discrepancies with the current findings may be attributable to differences in the measure of hunger, the age range of participants, or the time period in which the survey was conducted.

Approximately 7% of Inuit children experienced hunger regularly at the end of each month and about 5% experienced hunger more often than that. Budgets and food can become limited towards the end of the month (9). Additionally, food insecurity typically increases prior to pay day and during fall when ice and other weather conditions limit the ability to hunt (7).

Four out of five ever-hungry Inuit children had a parent/guardian who sought help from friends or family as a coping strategy for hunger. Other studies have also described this as a way of coping; the sharing of traditional foods is particularly important (7, 26). More than three-quarters of Inuit pre-schoolers lived in homes that shared traditional foods (15, 17). In this study, just over 1 in 10 ever-hungry Inuit children had parents/guardians who sought help from institutions as a coping strategy. Fewer resources may be available in low population density communities (27). Other strategies may include parents or children skipping meals (9, 17) – over 22% of ever-hungry children had parents/guardians who reported coping in this way. Other research has suggested that some may rely on selling assets (e.g. hunting gear, home items) to gain funds for purchasing foods, or that parents drink more coffee or tea to hide the hunger, or purchase the cheapest and most filling food items, such as pasta (7).

Dietary behaviours among Inuit children varied by hunger status: those who had ever experienced hunger drank less milk and milk products, and ate fish, eggs and meat, fruit and vegetables less frequently than those who had not experienced hunger. Other studies have found food insecurity to be related to lower prevalence of milk consumption (16) and fruit and vegetable consumption (28). Perishable foods including milk, fruit and vegetables are expensive and often of decreased quality in the North (7, 11). Ever experiencing hunger was not associated with consumption of fast foods or other high-fat/high-sugar foods and beverages. Other research from the U.K. (29) found no difference in the consumption of confectionary foods (e.g. chocolate, sweets) amongst 3-year-old children living in food-insecure and food-secure homes; however, in that study, those living in food-insecure homes consumed more soft drinks than those in food-secure homes.

The consumption of traditional foods has been positively associated with health (11), with the consumption of traditional food being associated with a more positive nutrient intake (e.g. greater protein and omega-3 fatty acid) for both adults (30) and children (14). The prevalence of consuming traditional foods was higher among children reported to be ever-hungry than never-hungry. For instance, consumption of bannock was twice as likely among ever-hungry children than among never hungry children. Some have argued that bannock may be a dietary staple when Inuit families have little to no money left (7), suggesting that income, rather than traditional food consumption itself, may be driving a relation between traditional food and hunger. Moreover, traditional food consumption may depend on geographic factors, including northern latitude (14), population density (31), or proximity to a coastal region (32), thus confounding the association between hunger and traditional food consumption.

It is also possible that areas in which traditional foods are more commonly consumed are also areas in which access to healthy foods is less plentiful or where hunger is more prevalent, suggesting a spatial effect. In the final logistic regression model, consumption of traditional foods was not associated with hunger status once Inuit region (and income) was considered. The prevalence of hunger was higher in Inuit Nunangat than outside Nunangat. Subsequent analyses also demonstrated that traditional food consumption was higher in the Inuit Nunangat regions. Further work on availability and cost of specific foods in Inuit Nunangat, and associations with traditional food consumption as well as hunger, is warranted.

The relationships between hunger and many of the covariates examined in our study are consistent with other studies, specifically decreased odds of experiencing hunger with increasing household income and home ownership, and increasing odds with increasing household size (16, 33, 34). Residing in the Inuit regions was associated with increased odds of experiencing hunger for Inuit children compared to residing outside Inuit Nunangat. The physical environment and isolated location of communities in the North may influence access to food (11), possibly differentially across Inuit regions (32). However, poverty restricts access even when food is available (11). Recent estimates suggest the average weekly cost of a food basket in remote northern communities is significantly higher compared to a food basket purchased in southern Canadian cities; for example, a food basket in Pond Inlet was $380 in 2006, compared to $206 in Ottawa (10). Some programs have been implemented to provide enhanced access and availability to quality foods in the North, specifically Nutrition North Canada, with the intent to provide higher subsidies for healthier foods and lower subsidies for less nutritious foods (35).

Living in a community that was rated by the parent as having cultural activities was associated with lower odds of experiencing hunger once all other factors were considered. This may relate to the parent-reported strategy of coping with hunger by seeking help from family or friends as it is possible that cultural activities (e.g. community feasts) include food, especially traditional foods. Furthermore, sharing of country food has been suggested as an important deterrent to food insecurity (8).

This study is the first nationally representative study to examine hunger for Inuit children in Canada, in particular with regards to diet composition and correlates of hunger. However, a number of limitations should be identified. Due to the cross-sectional nature of the data, cause-and-effect relationships cannot be determined. Having a single indicator to assess child hunger may have led to a misclassification of hunger status. That is, it is possible that a single indicator does not capture all children who had experienced hunger. In addition, a single indicator is not necessarily the same as a measure of food insecurity. Also, a reference time frame is not provided; the question simply asks if the child has “ever been hungry”. To assess nutrition intake, the 2006 ACS provided a non-exhaustive list of dietary items, and although frequency of consumption of these food items was assessed, information was not collected on the quantity of the food consumed. A more rigorous means of collecting information on food and beverage consumption would be with a 24-hour dietary recall. All outcomes were reported by the parent/guardian. Since many preschool-aged Inuit children use non-parental childcare arrangements (36), multiple sources of information would provide a clearer picture of what foods children are consuming in different environments. Questions on access to foods or the availability of support networks were not included in the ACS. Finally, the data was collected during the winter months which may have affected the availability of certain foods, in particular traditional or country foods or fruit and vegetables, and may have underestimated consumption.

In conclusion, almost a quarter of Inuit children in Canada are reported by parents to have experienced hunger. Ever-hungry children were reported to consume milk and milk products; fish, eggs and meat; fruit; and vegetables less often than never-hungry children; but fast food and processed foods, soft drinks and juice, and salty snacks, sweets and desserts as often as never-hungry children. The majority of ever-hungry Inuit children had parents/guardians who sought help from family or friends for support. However, factors associated with an increased likelihood of experiencing hunger for Inuit children include sociodemographic characteristics (e.g. lower household income) and residing in an Inuit region, whereas living in a community with cultural activities was associated with a decreased likelihood of ever having experienced hunger. Future research could further examine detailed food diaries, coping strategies, the role of the community and the impact of ever experiencing hunger on the health status of Inuit children and their families in Canada.

## Footnotes

1 Bannock is an Aboriginal country food, in the grains food group, that was introduced through early interactions with Europeans, likely Scottish fur traders (25).
